# Quantifying the role of chaperones in protein translocation by computational modeling

**DOI:** 10.3389/fmolb.2015.00008

**Published:** 2015-03-23

**Authors:** Salvatore Assenza, Paolo De Los Rios, Alessandro Barducci

**Affiliations:** Laboratoire de Biophysique Statistique, Ecole Polytechnique Fédérale de LausanneLausanne, Switzerland

**Keywords:** translocation, Hsp70, chaperones, molecular dynamics, free energy

## Abstract

The molecular chaperone Hsp70 plays a central role in the import of cytoplasmic proteins into organelles, driving their translocation by binding them from the organellar interior. Starting from the experimentally-determined structure of the *E. coli* Hsp70, we computed, by means of molecular simulations, the effective free-energy profile for substrate translocation upon chaperone binding. We then used the resulting free energy to quantitatively characterize the kinetics of the import process, whose comparison with unassisted translocation highlights the essential role played by Hsp70 in importing cytoplasmic proteins.

## 1. Introduction

Molecular chaperones are protein machines that assist other proteins in various cellular processes. 70-kDa Heat Shock Proteins (Hsp70s) are possibly the most versatile chaperones, supervising a wide variety of cellular tasks (Mayer and Bukau, 2005) that range from disaggregation of stable protein aggregates (Diamant et al., 2000) to driving post-translational import of cytoplasmic proteins into organelles (Matlack et al., 1999; Neupert and Brunner, 2002; Liu et al., 2014). Notably, Hsp70s play a fundamental role in the import of proteins into mitochondria because the majority of mitochondrial proteins are actually encoded in nuclear DNA, synthesized in the cytosol and only post-translationally imported into the organelles. Protein import takes place through a proteinaceous pore that spans the two mitochondrial membranes by way of the outer (TOM) and inner (TIM) membrane pore complexes (Neupert and Brunner, 2002). According to the current view, an ATP-consuming import motor located into the mitochondrial matrix drives the inward translocation of nuclear-encoded proteins. Mitochondrial Hsp70 (mtHsp70) is the central element of this motor: it is recruited by the TIM complex on the matrix side through interactions with the TIM44 protein, which is part of the pore, and with the pore-associated PAM16/18 proteins. The latter contain a J domain, whose role is to dramatically enhance the ATP-hydrolysis rate of Hsp70, thus increasing by orders of magnitude its affinity for substrates. The ATP-driven binding of the chaperones to incoming proteins ultimately drives their translocation.

**Figure 1:**
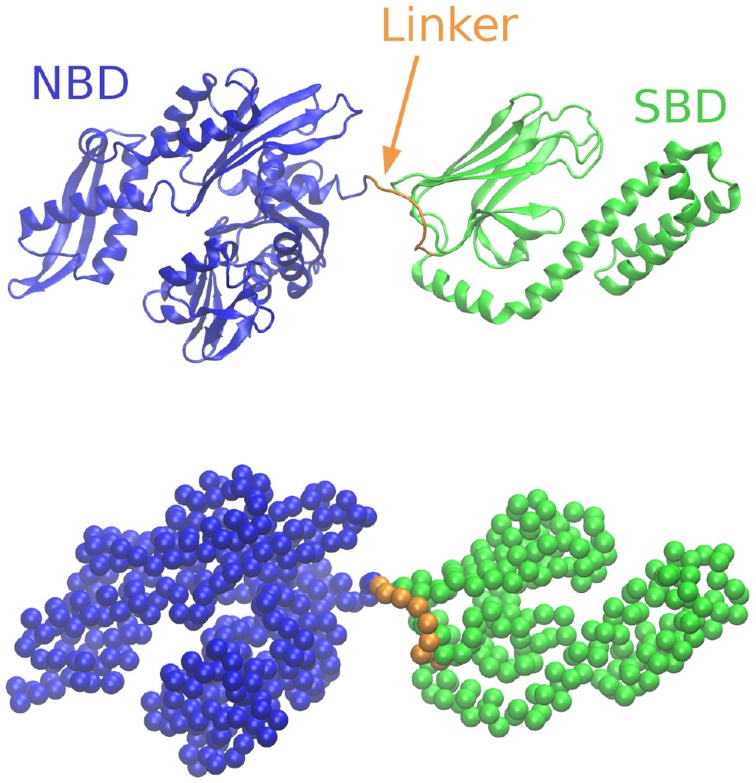
**Cartoon representation of the Hsp70 chaperone at the secondary-structure level (top) and corresponding coarse-grained model considered in this work (bottom)**.

The structure of Hsp70 is highly conserved (Zuiderweg et al., 2013) and consists of two large domains connected by a small flexible linker (see Figure 1). Specifically, the Nucleotide Binding Domain (NBD) is the ATPase unit of the chaperone, while the Substrate Binding Domain (SBD) directly interacts with specific sites on the incoming protein. These binding sites are frequently found in protein sequences, so that multiple chaperones are likely to bind the same substrate.

The precise mechanism by which Hsp70 exerts its pulling action has been debated in the literature and several models have been proposed (Glick, 1995; Neupert and Brunner, 2002; De Los Rios et al., 2006). The *Brownian ratchet* (Neupert and Brunner, 2002) assumes that, thanks to the chaperone large size, Hsp70 binding prevents the retrotranslocation of the substrate into the pore, thus biasing the random fluctuations toward the matrix. Alternatively, according to the *power stroke* (Glick, 1995) the chaperone actively pulls the incoming protein by using TIM as a fulcrum. Later, according to the *entropic pulling* model (De Los Rios et al., 2006), it was shown that an active force naturally emerges from a realistic physical description of the Brownian ratchet, thus reconciling the two views (Goloubinoff and De Los Rios, 2007). Indeed, the excluded volume of the chaperone, besides preventing retrotranslocation, reduces the conformational space available to the incoming protein, thus decreasing its entropy. This reduction depends on the length of the imported fragment of the substrate, therefore resulting in a free-energy gradient which favors the import.

In the present work, we evaluate this thermodynamic force in an effective one-dimensional space where the state of the system is represented by the number *n* of imported residues. In order to do so, for each value of *n* we compute the effect of chaperone binding on the free energy of the system by means of coarse-grained Molecular Dynamics (MD) simulations. This result is then used to devise a simplified yet quantitative analysis of the import, described as a one-dimensional diffusion process on the computed free-energy landscape.

## 2. Materials and methods

### 2.1. Details of MD simulations

We coarse-grained both the substrate and the chaperone by considering one interaction site per residue centered on the C_α_ atom. Residue-residue excluded-volume interactions were modeled with a repulsive Lennard-Jones potential with parameters σ = 3.8 Å and ε = 3*k_B_T*. The substrate was modeled by using the local flexible potential introduced in Ghavami et al. (2013). Within this force field, the elastic properties of a coarse-grained unfolded protein are described by means of harmonic C_α_ - C_α_ bonds and sequence-specific bending and torsional potentials. Particularly, the sequence dependence is introduced by considering a simplified three-letters description, where the eventual presence of glycine or proline amino acids is explicitly accounted for, while all the other residues are considered equivalent. In the present work, for simplicity we focus on glycine- and proline-free substrates, thus making use of the functions denoted as O-X-Y and X-X in Ghavami et al. (2013) for the bending and torsional contributions, respectively. The experimental structure of ADP-bound Hsp70 (Bertelsen et al., 2009) (PDB: 2KHO) was used to model the chaperone. In particular, the NBD (residue 4–387) and SBD (residue 397–603) were treated as rigid bodies, while the flexibility of the interdomain linker was accounted for by means of the potential described above. In order to reproduce a correct chaperone-substrate arrangement, we took advantage of the substrate-bound X-ray structure of DnaK SBD (PDB: 1DKX Zhu et al., 1996). MD simulations were performed using LAMMPS code (Plimpton, 1995) at constant temperature (*T* = 300 K) by means of a Langevin thermostat with damping parameter equal to 100 fs and using an integration timestep of 10 fs. For each value of *n* in the range 8 ≤ *n* ≤ 26 we performed a MD simulation of 5 · 10^10^ timesteps (examples of the convergence of the ratio 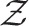
_70_(*n*)/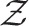
(*n*) are reported in the Figure S1—Supplementary Material), and the error on the free-energy profile was estimated by block averaging (Frenkel and Smith, 2002).

### 2.2. Details of the stochastic simulations

The import process was simulated by means of a Monte Carlo (MC) algorithm driven by the free-energy landscape *F*_import_, as determined from the sum of the chaperones pulling contribution computed by means of the MD simulations and the unfolding free energy *F_u_*. The latter is modeled as a sigmoidal function

$$F_{u}(n_{\text{in}})=\frac{F_{u}^{\text{max}}}{1+\exp\left[5-10(n_{\text{in}}-10)/\delta n\right]},$$

where *n*_in_ is the total number of imported residues, and *F*^max^_u_ and δ*n* are tunable parameters representing the total free energy of unfolding and the cooperativity of the unfolding process, respectively (see **Figure 4** top). For a system at position *n*_in_, a trial move was attempted to either *n*_in_ + 1 or *n*_in_ − 1 with equal probability and accepted according to the Metropolis criterion based on the free energy *F*_import_. To capture the sequence heterogeneity of the proteome, for each choice of *F^max^_u_* and δ*n* we generated 25 independent binding-site distributions, with the sole prescription that the average distance between consecutive binding sites was 35 residues as indicated by experiments (Rüdiger et al., 1997). For every distribution we performed 10 independent realizations of the import process. Average import times were estimated by counting the total number of MC timesteps needed for the translocation process to be completed. This protocol is justified by the fact that MC simulations correspond to overdamped Langevin dynamics when only local moves are considered (van Kampen, 1992; Tiana et al., 2007). Rescaling the obtained import times by the acceptance rate, as proposed in Sanz and Marenduzzo (2010), did not affect the results, because of the large fraction of accepted moves observed in all the simulations (> 95%).

## 3. Results

Protein import into organelles has been previously modeled as a one-dimensional stochastic process in the space of the imported residues (Elston, 2000, 2002; Liebermeister et al., 2001). In the present context, this protocol is justified by the timescale separation among substrate conformational dynamics, chaperone binding/unbinding and overall import. Indeed, the typical reconfiguration time of an unfolded protein (~ 100 ns Soranno et al., 2012) is extremely fast compared to the experimentally-determined timescale for protein import into mitochondria (order of minutes Lim et al., 2001). Effects arising from substrate conformational dynamics, such as the chaperone-induced entropy reduction, can be thus conveniently represented as effective free-energy profiles influencing the import dynamics. Moreover, the import timescale is also significantly slower than chaperone binding but faster than chaperone unbinding at physiological conditions. Indeed, according to the current understanding of the biochemical cycle of Hsp70 (Zuiderweg et al., 2013; De Los Rios and Barducci, 2014), ATP-bound chaperones associate with the substrate with a timescale equal to ~ 10^−2^ s (as estimated from a Hsp70-peptide association rate equal to 4.5 × 10^5^ M^−1^s^−1^ Schmid et al., 1994 and a chaperone concentration of 70 μM in mitochondria Liu et al., 2003), while dissociation takes place from the ADP-bound state, over timescales ~ 10^3^ s (Mayer et al., 2000). In the mitochondrial matrix chaperone-substrate dissociation is accelerated by the presence of nucleotide exchange factors, that catalyze the release of ADP and the ensuing rebinding of ATP. However, ATP hydrolysis is greatly enhanced in the proximity of the pore by pore-associated J-domain proteins, resulting into ultra-affinity for the substrate (De Los Rios and Barducci, 2014) and thus into an immediate replacement of the dissociated chaperone. This suggests that, to our purposes, we can assume that a chaperone immediately and irreversibly binds each exposed binding site as soon as it is imported. As a consequence, for the present purposes the number *n* of substrate residues that have been imported into the mitochondrial matrix is a convenient coordinate to describe the system, whose dynamics can be modeled as a diffusion process on the corresponding free-energy landscape.

### 3.1. Free energy calculation

**Figure 2:**
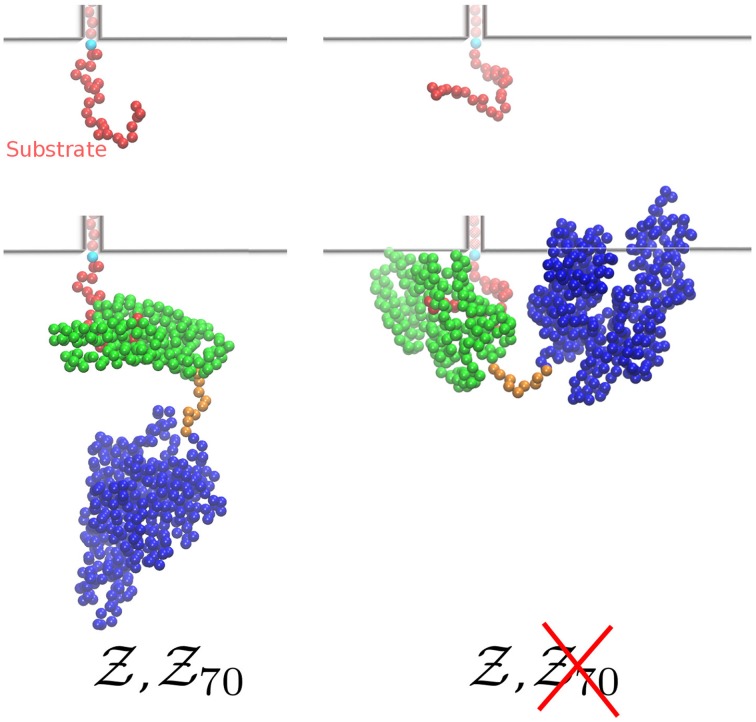
**Two representative conformations of the substrate without (top) and with (bottom) bound chaperone**. While in the absence of Hsp70s both conformations contribute to 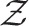
(*n*), upon chaperone binding the one on the right is not taken into account in 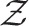
_70_(*n*) due to sterical clash with the wall. The *n*th residue of the substrate, which is constrained on the wall, is colored in cyan. The shaded beads inside the channel are here drawn only for representative purposes.

**Figure 3:**
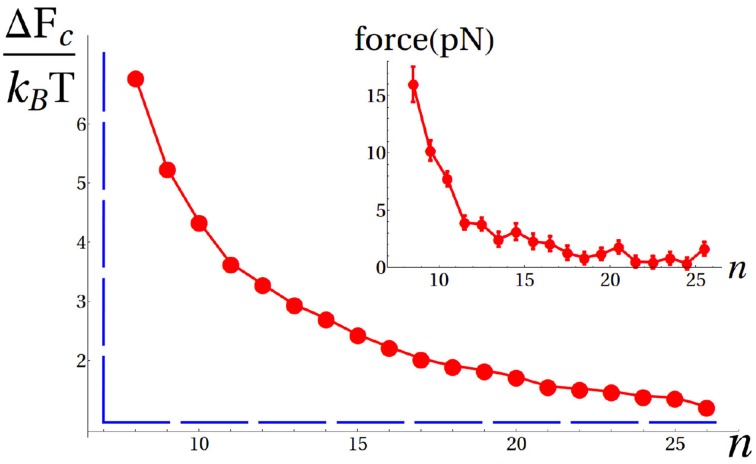
**Free-energy profile due to chaperone binding as a function of *n* (red circles)**. Error bars are smaller than the size of the symbols. The dashed blue line depicts the free-energy landscape predicted by the original Brownian ratchet, where the only effect of the chaperone is to prevent retrotranslocation beyond the binding site (infinite wall). In the inset we report the thermodynamic force corresponding to the computed free-energy landscape.

The effect of the size of the chaperone is two-fold. On the one hand, bound Hsp70 prevents the retrotranslocation of the substrate beyond its binding point (Brownian ratchet model Neupert and Brunner, 2002). On the other hand, the size of the chaperone leads also to a reduced number of sampled conformations (entropic pulling De Los Rios et al., 2006), an effect not accounted for by the Brownian ratchet as it was originally conceived, but nonetheless intimately related to the same physical mechanism. For example, in the absence of Hsp70 the two substrate conformations shown in the top panel of Figure 2 are both sterically allowed. However, upon chaperone binding the conformation on the right would result into an overlap between the membrane and Hsp70 (bottom panel in Figure 2), and it is therefore never sampled by the substrate when the chaperone is present. The free energy difference due to the loss of entropy is given by Δ*F_c_*(*n*)= −*k_B_T*log(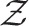
_70_(*n*)/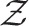
(*n*)), where 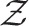
_70_(*n*) and 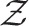
(*n*) are the partition functions of the substrate with and without a bound chaperone, *k_B_* is the Boltzmann constant and *T* the temperature (when enthalpic contributions are not taken into account, the partition functions reduce to the number of sampled conformations, thus falling back to the original formulation of the entropic-pulling free energy De Los Rios et al., 2006). Here, we computed the free energy difference Δ*F_c_*(*n*) by estimating the ratio 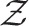
_70_(*n*)/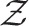
(*n*) for *n* in 8 ≤ *n* ≤ 26 with multiple coarse-grained MD simulations. The substrate was modeled as a *n*-residues flexible chain with the position of the *n*th residue constrained on the inner mithocondrial membrane, represented here as a flat wall acting only on the substrate residues (see Materials and Methods for additional details). As a consequence, the system could sample configurations involving an overlap between the membrane and the chaperone (see bottom-right panel in Figure 2). With this strategy, we could estimate the ratio 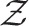
_70_(*n*)/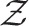
(*n*) as the fraction of time spent by the system in physically-acceptable, i.e., non-overlapping, configurations. Particularly, we focused on *n* ≥ 8 in order to allow the exposure of a complete binding site. From the computed values of 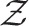
_70_(*n*)/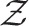
(*n*), we could retrieve the free energy Δ*F_c_*(*n*) as a function of *n*, as reported in Figure 3. As expected, shorter imported fragments resulted into a larger fraction of rejected conformations, i.e., larger values of Δ*F_c_*, thus leading to a free-energy gradient favoring the import of the protein. The slope of the entropic-pulling free-energy profile corresponds to the thermodynamic pulling force exerted by a bound chaperone along *n* (Figure 3 inset). This force is in the piconewton range, starting from around 15 pN and decreasing as *n* increases. Remarkably, these results agree qualitatively with previous estimates based on strongly simplified representations of the system (De Los Rios et al., 2006), thus suggesting that comparable thermodynamic forces could be obtained by the same entropic pulling mechanism for macromolecules of similar size.

### 3.2. Stochastic simulations of the import process

**Figure 4:**
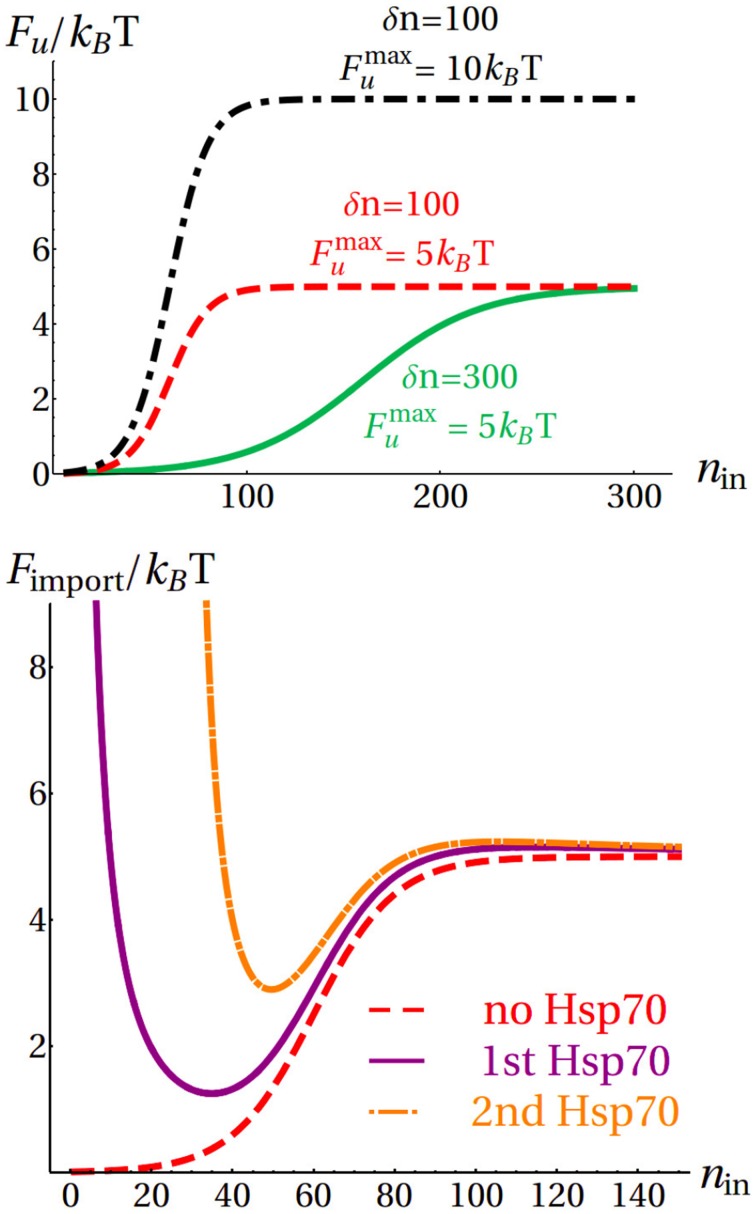
**Top:** Influence of the parameters *F*^max^*_u_* and δ*n* on the unfolding free-energy. **Bottom**: Evolution of the total free-energy *F*_import_ in a representative import process.

We modeled the import of cytoplasmic proteins as a one-dimensional stochastic process depending on the number *n*_in_ of imported amino acids. The effective free-energy profile guiding the system evolution results from protein unfolding (Eilers and Schatz, 1986) and active chaperone pulling (Lim et al., 2001). Assuming a two-states folding behavior, a convenient choice to model the unfolding contribution to the free-energy landscape is a tunable sigmoidal function *F_u_*(*n*_in_) (see Materials and Methods), depending on two parameters that measure the total unfolding free energy (*F*^max^*_u_*) and the cooperativity of the unfolding process (δ*n*), with smaller values of δ*n* corresponding to higher cooperativity (top panel in Figure 4). By tuning these parameters, the formula can account for the wide variety of imported proteins (Wilcox et al., 2005). The pulling action of the chaperone was modeled taking advantage of the free-energy profile determined from molecular simulations. Particularly, we assumed here that: (i) Hsp70s associate with each binding site as soon as it emerges from the pore, since they are targeted at the TIM pore exit by specific interactions (Neupert and Brunner, 2002); (ii) we considered only the contribution arising from the Hsp70 closest to the pore, taking into account the relatively fast decrease of the slope of Δ*F_c_* (see Figure 3) and the average frequency of binding sites (one every 35 amino acids Rüdiger et al., 1997). Therefore, we added to the unfolding free-energy *F_u_*(*n*_in_) the chaperone contribution Δ*F_c_*(*n*_in_ − *n_B_*), with *n_B_* corresponding to the position of the binding site closest to the pore, measured from the matrix terminus of the substrate.

As an example, in the bottom panel of Figure 4 we illustrate the evolution of the free-energy landscape during the import process of a protein with *F*^max^*_u_* = 5*k_B_T*, δ*n* = 100 and two binding sites at *n_B_* = 0 (i.e., at the matrix terminus) and *n_B_* = 28. At the beginning of the import process, no chaperone is bound to the substrate and the import free energy is simply given by *F*_import_(*n*_in_) = *F_u_*(*n*_in_) (red dashed curve). As soon as the first binding site is imported, a chaperone molecule binds the substrate and its contribution Δ*F_c_* is added to *F_u_*(*n*_in_) starting from the binding site *n_B_* = 0: *F*_import_(*n*_in_) = *F_u_*(*n*_in_) + Δ*F_c_*(*n*_in_) (purple continuous curve). Finally, after the second binding site (*n_B_* = 28) is imported, another chaperone binds and the resulting free energy is *F*_import_(*n*_in_) = *F_u_*(*n*_in_) + Δ*F_c_*(*n*_in_ − 28) (orange dot-dashed curve).

**Figure 5:**
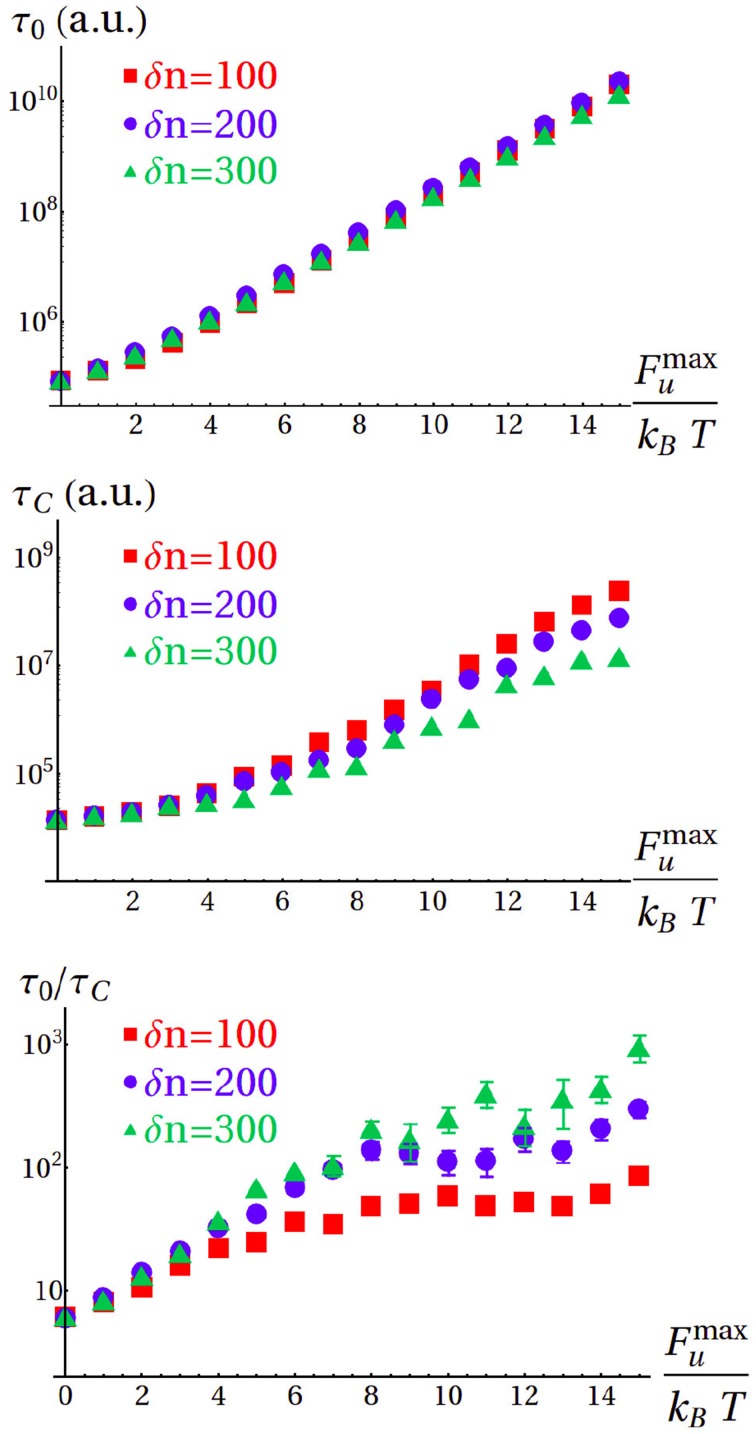
**Top:** Average import times in the absence of chaperone (τ_0_) as a function of *F*^max^*_u_* for different cooperativities (values for *F*^max^*_u_* ≥ 12*k_B_*T were extrapolated by fitting the data in the range 4*k_B_T* ≤ *F*^max^*_u_* ≤ 11*k_B_T* with exponential functions). **Center**: Average import times in the presence of Hsp70 (τ_*C*_) for the same cases as in the top panel. **Bottom**: Acceleration of the process due to the assistance of Hsp70, expressed as the ratio τ_0_/τ_*C*_.

Following this approach, we computed the average import time (see Materials and Methods) of 300-residue proteins for different values of δ*n* and a range of *F*^max^*_u_* corresponding to the stability of a large fraction of the proteome (Ghosh and Dill, 2010). In absence of Hsp70 assistance, the system must invariably overcome a free-energy barrier, and the average import time τ_0_ increases exponentially with *F*^max^*_u_*, independently of cooperativity (Figure 5 top). In all the considered cases, the average import time for the chaperone-assisted process, τ_*C*_, is sensibly smaller than τ_0_ (Figure 5 center). The chaperone pulling force reduces but does not completely eliminate the unfolding free-energy difference for stable proteins (large *F*^max^*_u_*), as in the case of the representative process shown in the bottom panel of Figure 4. In this regime, the import is still an activated process, and the average times increase exponentially with *F*^max^*_u_*. Conversely, the pulling action of Hsp70 dominates over the unfolding contribution for marginally stable proteins (small *F*^max^*_u_*), thus resulting in values of τ_*C*_ comparable to what found for the extreme case *F*^max^*_u_* = 0. The import kinetics is further modulated by δ*n*, with high cooperativity (small δ*n*) resulting in longer translocation times.

In the bottom panel in Figure 5 we illustrate the chaperone-induced kinetic advantage by reporting the ratio τ_0_/τ*_C_*. This ratio ranges from a 10-fold gain for marginally stable proteins to 10^3^ for extremely stable and non-cooperative substrates, with the majority of the proteome (*F*^max^*_u_* ≥ 8*k_B_T* Ghosh and Dill, 2010) accelerated at least 100 times. If we take into account that protein import into mithocondria has been measured to happen in the timescale of several minutes (Lim et al., 2001), our model indicates that the translocation process in the absence of chaperones would probably extend to hours or days. Since such a slow process would clearly be incompatible with the average lifespan of proteins and the duration of the cell cycle, our results provide a molecular basis to support the essential role of chaperones in the *in vivo* import process.

## 4. Conclusions

To summarize, in this work we derived a free-energy profile for the import process based on a molecular description of Hsp70 that rationalizes the requirement for chaperone assistance in mitochondrial protein import observed in experiments. Naturally, a more precise quantitative estimation could be obtained by considering a more refined representation of the system, i.e., going beyond the coarse-grained model at residue level resolution employed in this work and considering also other interactions than the excluded volume. The present results can be applied to other cases of Hsp70-driven translocation, namely protein import into ER (Matlack et al., 1999) and chloroplasts (Liu et al., 2014). In the ER case, in particular, the pore is much simpler than in mitochondria, as it spans just a single membrane. The presence of Sec63, a pore-associated protein containing a J domain, ensures also in this context that the extended ATP-driven Hsp70 ultraffinity prevails in the competition against other translocation counterproductive interactions (Scidmore et al., 1993). Moreover, this approach based on the combination of molecular simulations and kinetic modeling can be easily extended to other Hsp70-mediated cell processes. In particular, this free-energy picture could help to understand some recent results pointing toward a fundamental role of Hsp70 in preventing the stalling of translation at ribosomes (Liu et al., 2013; Shalgi et al., 2013). Owing to the universality of the interaction responsible for the effects studied here, namely excluded volume, the same principles could apply to similar processes driven by other biomolecules.

## Author contributions

SA, PDLR, and AB designed and performed research, analyzed the results and wrote the paper.

## Conflict of interest statement

The authors declare that the research was conducted in the absence of any commercial or financial relationships that could be construed as a potential conflict of interest.

## Supplementary material

Supp. Figure 1 **Evolution of the ratio _70_(*n*)/(*n*) as a function of MD timesteps, for *n* = 7 (top panel), *n* = 16 (middle panel) and *n* = 25 (bottom panel)**.
