# Supplementary figures and images for: Quantifying the role of chaperones in protein translocation by computational modeling
Source: Front Mol Biosci. 2015 Mar 23;2:8. doi: 10.3389/fmolb.2015.00008 (PMC4428437; doi:10.3389/fmolb.2015.00008)

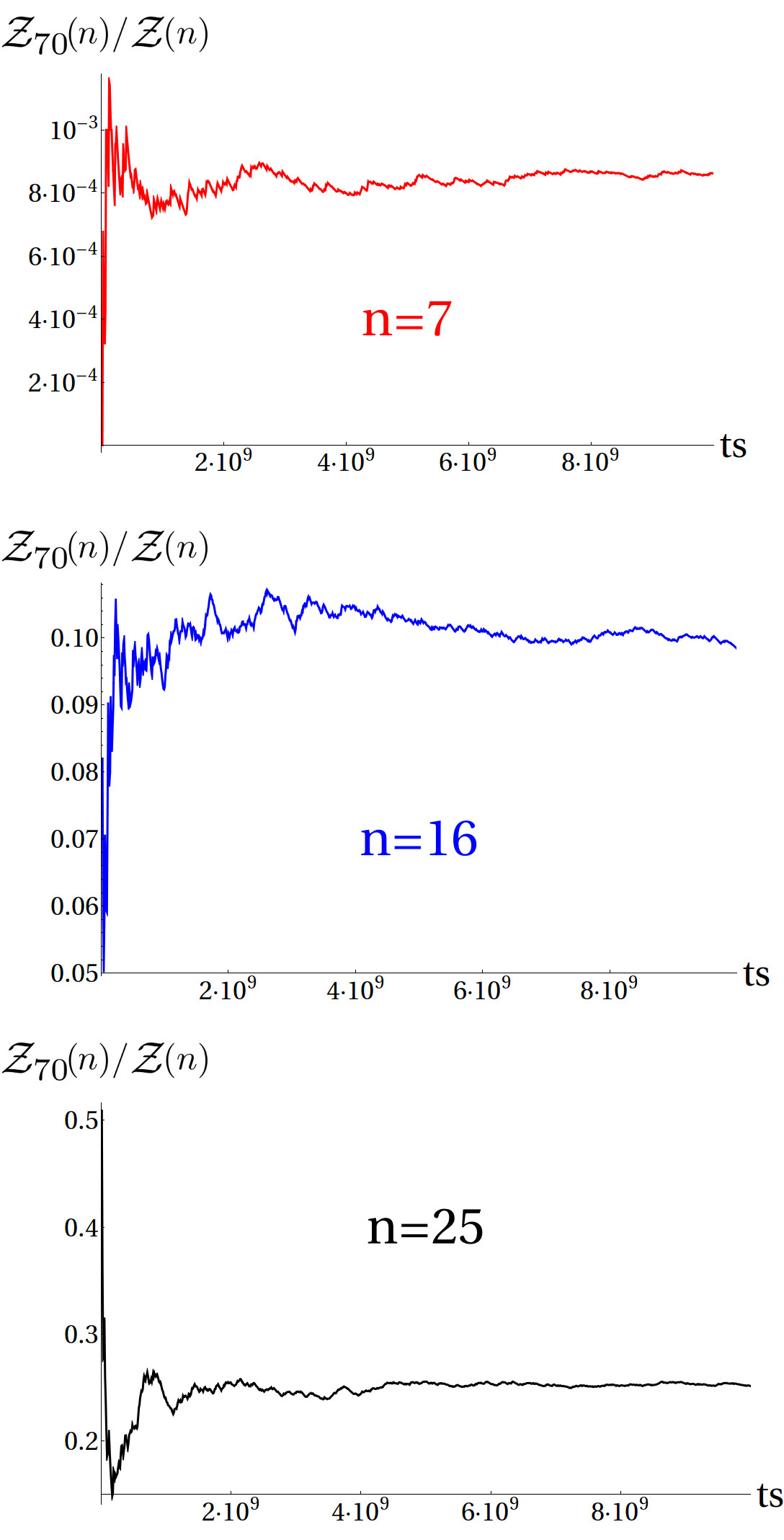

Supplement: Supp. Figure 1 — Evolution of the ratio 70(n)/(n) as a function of MD timesteps, for n = 7 (top panel), n = 16 (middle panel) and n = 25 (bottom panel). [file Image1.JPEG]
